# Monarch performance and cardenolide sequestration decline on naturally derived hybrid milkweeds (Asclepias syriaca x speciosa)

**DOI:** 10.21203/rs.3.rs-9919052/v1

**Published:** 2026-06-15

**Authors:** Carrie F. Olson-Manning, Sneha Acharya, Lara Matuck, Gabriela Rabboni, Madelyn Sliper, Scott Shlanta, Andrew Berntson, Jacob Mills, Daniel Street, Beau Brock, Lilian Derynck, Kiera Kuiper, Steven L. Matzner, Sydney N. Muller

**Affiliations:** Augustana University; Augustana University; Louisiana State University; Augustana University; Augustana University; Augustana University; Augustana University; Tulane University; Augustana University; Augustana University; Augustana University; Augustana University; Augustana University; Augustana University

## Abstract

Plant hybridization often alters herbivore-relevant traits, yet the effects of hybrid feeding on specialist herbivores, especially those that co-opt host-plant defenses, are largely unknown. We examined how naturally derived hybrids between *Asclepias syriaca* and *A. speciosa* (*A. syriaca x speciosa*) affect monarch butterfly (*Danaus plexippus*) performance and cardenolide sequestration. Across three independent greenhouse experiments, monarch caterpillars reared on genetically heterogeneous *A. syriaca x speciosa* hybrid milkweeds grew more slowly than caterpillars reared on either parental species. Monarchs reared on hybrids also, on average, sequestered lower total cardenolide concentrations into adult tissues. However, plant measures of cardenolide chemistry did not differ significantly among parental and hybrid host groups, and measured physical defenses (trichome and latex) showed only partial differences among groups, and none in the expected direction. Element analyses of plant leaf tissue, although preliminary, revealed a marginally significant negative relationship between caterpillar growth and manganese level, with hybrids containing between two- to three-fold higher levels of manganese as either parental species. These results indicate that host-plant hybridization can alter both specialist herbivore performance and acquired chemical defense, even when broad measures of host defenses do not explain those effects. Hybridization may therefore influence plant-herbivore interactions not only by changing host quality, but also by altering how specialist herbivores acquire plant-derived defenses.

## INTRODUCTION

Specialist herbivores have co-adapted physiological and behavioral processing to overcome host-plant defenses. However, hybridization between plant species often reshapes defensive traits causing diverse, often non-additive consequences for insect herbivores (Fritz *et al*. 1999; López-Caamal and Tovar-Sánchez 2014; Porretta and Canestrelli 2023). Hybrid plants can have intermediate or transgressive trait expression or novel combinations of traits compared to the parental species (Kuzina *et al*. 2011; Kirk *et al*. 2012; Caseys *et al*. 2015). Because specialist herbivores often rely on tightly coevolved systems with a range of host-specific behavioral and physiological adaptations (Fürstenberg-Hägg *et al*. 2013; Dussourd 2017; War *et al*. 2018; Abbas *et al*. 2025), this reshuffling of trait combinations can be particularly impactful.

Plant hybridization can break down the co-evolutionary arms race between the physical and chemical defenses of host plants and corresponding specialist herbivore adaptations. Physical barriers, including trichomes and latex exudation, are overcome with specific feeding behavior and chemical defenses are selectively detoxified, excreted, or even co-opted by herbivores for their own defense (Beran and Petschenka 2022). The ability of an herbivore to access a plant can also be driven by adaptations of the plant to its abiotic environment (Carmona *et al*. 2011), resulting in complex, often synergistic action of uncorrelated traits. Thus, hybridization has the potential to make a host-plant more or less accessible to an herbivore compared to the progenitor species, even if both progenitors of the hybrid are available as host-plants to that hebivore (Hallgren 2003; Torp *et al*. 2013; Wei *et al*. 2015; Haireen *et al*. 2019; Renoult *et al*. 2025).

Perhaps the most well-studied model of specialist herbivore that co-opts its host plant’s defenses is that between *Danaus plexippus*, the monarch butterfly, and members of the milkweed family *Asclepiadoideae*. Monarchs feed exclusively on a wide variety of milkweed, primarily *Asclepias* species, and milkweeds have a set of physical and chemical defenses against monarch feeding (Malcolm and Brower 1989; Ladner and Altizer 2005; Agrawal *et al*. 2012). Trichomes prevent larvae from reaching leaf tissue and, once punctured, most milkweed species exude latex from their leaves, drowning up to 25% of monarch larva (Zalucki *et al*. 2001; Agrawal and Konno 2009). Cardenolides produced by milkweeds inhibit Na+/K+ ATPase pumps, disrupting cellular ion gradients and causing a myriad of neurotoxic effects (Agrawal *et al*. 2012). Monarchs, in turn, overcome these defenses by removing trichomes and cutting leaf veins, preventing latex exudation (Betz *et al*. 2024) and the caterpillars have specialized enzymes in their guts to digest harmful latex proteins and can expel cardenolides or detoxify them to less harmful forms (Pereira *et al*. 2010). Monarchs contain amino acid substitutions in their Na+/K+ ATPase pump proteins that prevent the worst effects of cardenolide cellular disruption (Holzinger *et al*. 1992; Holzinger and Wink 1996; Agrawal *et al*. 2012). The relationship between these specialized herbivores and the strong host plant defenses make the monarch-milkweed system ideal for understanding how co-evolutionary relationships may break down due to plant hybridization.

In addition to resistance and detoxification, monarchs have evolved the ability to sequester cardenolides for use in their own defense (Brower and Glazier 1975). The process of sequestration, a term that encompasses processes of selective absorption through the gut and reabsorption through the Malpighian tubes, biochemical modification, and transport within the body (Malcolm 1990; Beran and Petschenka 2022) is especially relevant as host chemistry becomes part of the herbivore defenses. These mechanisms are selective, and levels of sequestered compounds can depend on compound identity more than concentration in leaf tissue (Agrawal *et al*. 2026). Indeed, the sequestered compounds are not proportional to the composition found in plant tissues (Züst *et al*. 2019) and the distribution of the different cardenolides are different even among adult butterfly tissues (Brower *et al*. 1972).

The concentration, composition of the cardenolide variations, and transport to appropriate tissues are important for the protection of monarchs against their predators and parasites. Most famously, cardenolides are distasteful and emetic to avian predators causing birds to avoid future contact (Brower 1988). The concentration and localization of cardenolides of different emetic potencies are seen in the thorax, wings, and abdomen of adult butterflies, with the abdomen and wings as the most emetic (Brower *et al*. 1972). At an individual fitness level, higher concentrations of cardenolides diminish the size of protozoan parasites (*Ophryocystis elektroscirrha*) and the severity of infection (Gowler *et al*. 2015; Hoang *et al*. 2017; Hoogshagen *et al*. 2023). Parasitoid wasps will avoid oviposition in caterpillars with higher cardenolide concentrations (de Roode and Hunter 2019). Therefore, reduced sequestration could alter protection from enemies even when the survival or growth of the larva is unaffected by feeding on hybrid plants. However, despite the frequency of hybridization in plants (40–60% of plant families) (Whitney *et al*. 2010) and the hundreds of insect species known to sequester chemical defences from plants (Beran and Petschenka 2022), to our knowledge, few studies directly testing how hybrid host plants affect herbivore sequestration and performance.

To address this gap, we study two species of hybridizing milkweeds that are both preferred host plants of the monarch butterfly (Damiano and Perkins 2025). The milkweed species *Asclepias syriaca* (the common milkweed) and *Asclepias speciosa* (the showy milkweed) produce fertile hybrids (*A. syriaca x speciosa*) where their ranges overlap in the Great Plains of North America (Andreev et al. 2025). The geographic ranges of these milkweeds are vast, and their zone of contact stretches hundreds of miles, from the border of the United States and Canada to northern Oklahoma. The migratory routes of Eastern monarchs pass through this extensive hybrid zone (Reppert *et al*. 2010; Andreev *et al*. 2025), providing ample opportunity for monarchs to come in contact with *A. syriaca x speciosa* hybrids.

Here we measure monarch butterfly performance, through growth and sequestration, when caterpillars are reared on *Asclepias syriaca*, *A. speciosa*, or on genetically diverse *A. syriaca x speciosa* hybrids collected from across the region of sympatry. We also test whether any differences in performance are associated with differences in plant element abundance, leaf defensive traits, or cardenolide chemistry among parental genotypes and admixed genotypes. Consistent with a breakdown of herbivore-related traits in hybrid plants, monarchs reared on hybrid genotypes showed slower growth rates and higher mortality on average. As adults, hybrid-reared butterflies sequestered approximately two-fold lower concentrations of cardenolides. However, these differences in performance and acquired defense were not directly associated with plant cardenolide concentration or measured physical defenses. Instead, higher plant manganese concentrations were associated with slower caterpillar growth and reduced cardenolide sequestration, suggesting that reduced monarch performance on hybrid plants may be linked to altered micronutrient acquisition rather than to disrupted plant defenses alone.

## MATERIALS AND METHODS

### Plant growth conditions

In these experiments 12 *A. speciosa*, 10 hybrid, and 17 *A. syriaca* full-sibling families were collected from natural populations (Supplemental Table 1). Four F1 hybrid families were derived from a controlled cross in a common garden between *A. syriaca* and *A. speciosa* and one family was derived from a cross between two hybrid families. As described below, for each maternal family, one individual was sequenced and used to represent the estimate of ancestry of that family as milkweeds have a pollinarium-based pollination system meaning all seeds in a pod (denoted as family here) are full siblings.

Seeds were surface sterilized and stratified at 4 C for 30 days. Seeds were planted in either 38 mm × 209 mm inch conical pots or 89 mm × 127 mm pots in Promix BRK20 BioFungicide mycorrhizae soil supplemented with Osmocote fertilizer. Plants were grown in the greenhouse at Augustana University with an average temperature of 22.4 C and 16 hour grow light conditions.

### Ancestry determination

The ancestry of the families was determined following the genomic workflow described in Andreev et al. (2025). For genotyping, briefly, genomic DNA libraries were prepared using the PlexWell 96/384 library preparation kit (seqWell, Beverly, MA, USA) and sequenced at Michigan State University on an Illumina NovaSeq system with paired-end 150 bp reads and 96 samples per lane. Mean nuclear genome coverage was 4.3x. Reads were quality filtered, screened to retain nuclear reads and remove plastid and mitochondrial reads, and mapped to the *Asclepias syriaca* nuclear reference genome as described in Andreev et al. (2025). Variants were called with FreeBayes and filtered to retain high-quality nuclear SNPs using the following filtering criteria: SNPs were filtered to retain biallelic SNPs with quality score > 30, minor allele frequency > 0.05, mean genotype depth > 3, and ≤ 5% missing data per locus. The resulting SNP set was linkage disequilibrium-pruned using an r^2^ threshold of 0.2 in 1-kb windows. The final dataset contained 8,410 nuclear SNPs.

Individual ancestry was estimated from the filtered nuclear SNP dataset using fastSTRUCTURE (Raj *et al*. 2014). Each family was represented by one individual, and family representatives were assigned to *A. speciosa*, *A. syriaca*, or mixed ancestry when that family had less than 95% ancestry from one parental species or the other. The estimated proportion of the genome assigned to *A. syriaca* ancestry for each family representative is reported in Supplemental Table 1.

### Monarch growth conditions

Monarch butterfly (*Danaus plexippus*) eggs came from a colony established by Anurag Agrawal at Cornell University (for 2021 and 2023 experiments) or from a colony that was collected from natural populations near Sioux Falls, South Dakota and established at Augustana University (2025 experiment). Monarchs were raised in the greenhouse at an average temperature of 22.4 C and with 16 hour grow light conditions. To prevent acclimation to only one genotype caterpillars were grown on a rotating mixture of *Asclepias syriaca*, *A. speciosa*, and *A. syriaca x speciosa* hybrid leaves. For the 2021 and 2025 experiments the caterpillars were grown on intact plants. For the 2023 experiment, caterpillars were grown on either intact plants or on removed leaves. However, the caterpillar growth rates and adult sequestration levels of monarchs growth on cut leaf and intact plants did not differ (Welch Two Sample t-test, t = −1.14, df = 17.1, p = 0.268). This treatment was used as a random effect in subsequent linear mixed models, described below. Adult butterflies were fed 20% honey-water solution after emergence.

### Monarch caterpillar growth bioassays

Newly hatched *D. plexippus* caterpillars were placed on greenhouse-grown milkweed plants from both species of milkweed and genetically diverse hybrids until pupation. Caterpillar weight was recorded every other day for 10 days. All experiments had one caterpillar remain on one individual plant for the 10 days assay and until pupation.

### Leaf hair length and density imaging

Nine milkweed families from *A. syriaca*, nine from *A. speciosa*, and 16 from the *A. syriaca x speciosa* hybrids were planted in replicates of 10 each and watered every day until physical traits (trichomes and latex) were measured. Plants were allowed to grow for nine weeks and were selected for measurement once the plants were above 10 cm tall. The most recent fully expanded leaf was selected and the upper and underside of each leaf was imaged using a Canon camera (model EO5 Rebel T3) mounted on an Olympus CX21 light microscope under a 25x magnification (5x objective and 5x magnification). A 0.5 mm^2^ area on each image was selected with ImageJ in the middle of the leaf between leaf veins (leaf hair density was different along leaf veins). All trichomes completely inside the selected area were counted. Trichome length (mm) was measured from the bottom of each hair from the base to the tip.

### Latex exudation

Latex exudate levels were measured on the same nine-week-old milkweed plants as described above by cutting 0.5 cm off the tip of one of the upper fully expanded leaves. A small pre-weighed filter paper punch was held against the cut for 10 seconds to collect latex exudate and filter papers were weighed.

### Plant nutrient analysis

One to three individuals from eight *A. syriaca*, four *A. speciosa*, and five *A. syriaca x speciosa* hybrid families were grown for 3 months before leaf tissue was collected for nutrient analysis. Approximately 30 g of leaf tissue were removed from the plant, patted dry and stored in a paper bag and shipped on ice to Midwest Laboratories in Omaha, NE for a Plant Tissue Complete Analysis. Midwest Laboratories standardized the samples by dry weight prior to analysis. The elements measured are summarized in Table 1 and Supplemental Fig. 1. Units are in either % rate (Nitrogen, Phosphorus, Potassium, Magnesium, Calcium, Sulfur, Sodium, and Carbon), ppm (Iron, Manganese, Boron, Copper, Zinc), or a ratio of Carbon to Nitrogen.

Plant trait data were summarized at the family level prior to analysis to avoid pseudoreplication among individuals from the same family. For each family, we calculated the mean value of each measured trait and the mean percent *A. syriaca* ancestry. Family-level means were used as the units of analysis in all subsequent tests.

For each trait, we fit a linear model with family mean trait value as the response variable and genotype class as the predictor. We evaluated the overall effect of genotype using analysis of variance for each trait-specific model. To account for multiple testing across traits, p-values from the genotype terms were adjusted using the Benjamini-Hochberg false discovery rate procedure. Genotype-specific estimated marginal means were calculated using emmeans, and pairwise comparisons among genotype classes were performed using Tukey-adjusted contrasts.

### High-performance liquid chromatography of leaf and butterfly tissues

Leaf cardenolides were extracted from the first fully-expanded leaf of 9-week old plants as in (Petschenka *et al*. 2023) and monarch cardenolides were extracted from fore-wing, thorax, and abdomens of adult butterflies. Briefly, up to 50 mg of freeze dried leaf or 10–15 mg butterfly tissue was ground to powder in a 1.5 mL tube containing 1.25 mL 100% HPLC-grade methanol spiked with 20 ug digitoxin. Following centrifugation, the supernatant from the leaf extracted was evaporated to dryness in a rotary vacuum, resuspended in 200 uL 100% methanol, and analyzed via High-Performance Liquid Chromatography (HPLC). Butterfly tissue underwent an additional de-fatting protocol as in (Agrawal and Hastings 2023) prior to evaporation to dryness.

Cardenolides were detected via HPLC on an Agilent 1100 Diode Array Detector system, separated on a Gemini C18 reverse-phased column (3 μm, 150 × 4.6 mm, Phenomenex). Samples were run at a constant flow of 0.7 mL/min of the following gradient of acetonitrile and HPLC-grade water: 0 to 2 min at 16% acetonitrile; 2 to 25 min from 16 to 70% acetonitrile; 25 to 30 min from 70 to 95% acetonitrile; 30 to 35 min at 95% acetonitrile; followed by 10 min reconditioning at 16% acetonitrile. Ultraviolet spectra of peaks from 200 to 400 nm. Peaks were called cardenolides if they displayed an absorption spectrum with a single maximum around 218–220 nm (Petschenka *et al*. 2023). The concentration of individual cardenolide peaks were calculated as digitoxin-equivalents per mg dried tissue.

### Statistical analyses

All analyses were conducted in R using tidyverse for data wrangling and visualization and lme4/lmerTest for linear mixed-effects models. Estimated marginal means and post hoc contrasts were obtained using emmeans. Unless otherwise noted, fixed effects were evaluated using Type III ANOVA with Satterthwaite or Kenward-Roger degrees-of-freedom approximations.

To test whether monarch caterpillar growth differed among host genotype groups (*A. speciosa*, *A. syriaca*, and genetically diverse hybrids), we analyzed relative caterpillar growth using a linear mixed-effects model with genotype group (*A. syriaca*, *A. speciosa*, hybrids) as a fixed effect and experiment (three greenhouse experiments) as a random intercept (relative caterpillar growth ~ genotype group + (1 | experiment)). We calculated the relative growth within each experiment by dividing each individual final weight on day 10 to the weight of the individual with the highest weight. For interpretation of coefficients, genotype was coded with hybrid as the reference level.

Plant and butterfly tissue chemistry were only collected in the 2023 experiment. For each plant sample, we calculated: total normalized cardenolide concentration (sum of plant cardenolide peak areas normalized by plant digitoxin peak area and plant dry mass), polarity index (weighted mean retention time of detected peaks, using peak-area proportions (Rasmann and Agrawal 2011)), and peak richness (number of detected cardenolide peaks with nonzero area). Polarity index summarises cardenolide toxicity in a sample as the more toxic, non-polar cardenolides have higher retention times. Thus, a higher polarity index indicates a more toxic sample of cardenolides. Total normalized concentration was log-transformed prior to analysis and a small constant added before log transformation to all values to avoid log(0). Species differences in plant defense chemistry were tested using linear mixed-effects models with genotype group as a fixed effect and FamilyID and cut or intact plant treatment random effects. Leaf cardenolide variation was also visualized with a heatmap (Supplemental Fig. 2).

Plant families with data for growth in 2023 and 2025, corresponding adult monarch cardenolide concentration, and Manganese concentration are summarised in Supplemental Table 2.

We quantified three leaf surface defense traits—latex exudation, trichome length, and trichome density—and tested for differences among host genotype groups (*A. speciosa*, hybrids, and *A. syriaca*) using oneway ANOVA, followed by Tukey-adjusted post hoc comparisons of estimated marginal means where appropriate.

Butterfly tissue chemistry analyses included wing, thorax, and abdomen samples. For each tissue, normalized cardenolide concentration, polarity index, and cardenolide richness was calculated as described above. To compare tissue types, we fit a linear mixed-effects model with genotype group and tissue as the fixed effects and FamilyID and cut or intact plant treatment random effects. Pairwise comparisons among tissue levels were valued using Tukey-adjusted contrasts from eemeans.

To calculate overall sequestration per individual, we summed the cardenolide concentration in the wing, thorax, and abdomen of each individual and used a mixed model with genotype group as the fixed effect and FamilyID and cut or intact plant treatment random effects. We also calculated the relative allocation among butterfly tissues by calculating the proportion of total sequenced cardenolides in each tissue (wing, abdomen, thorax).

## RESULTS

### Monarch caterpillars have reduced growth rate when reared on genetically diverse A. syriaca x speciosa hybrids as compared to the parental species

**Figure 1:**
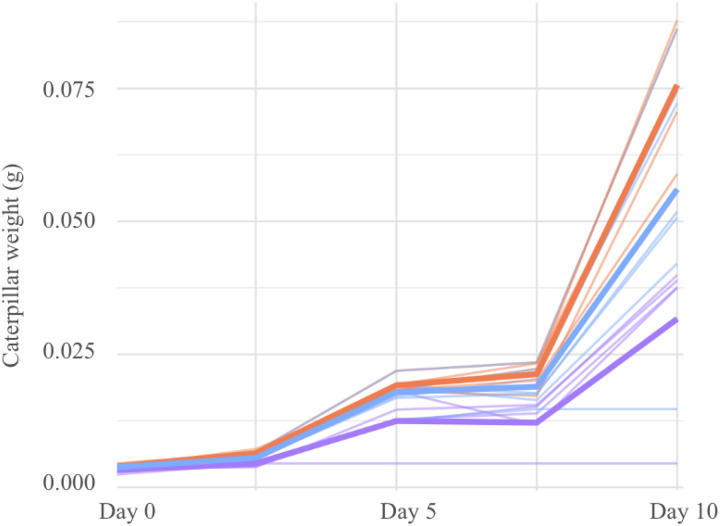
Monarch caterpillar growth measured in caterpillar weight over 10 days in the 2023 experiment. Growth of monarch caterpillars on greenhouse grown parental milkweed species (orange; *A. speciosa*, blue; *A. syriaca*) and on genetically diverse hybrids between the species (purple; *A. syriaca x speciosa)* in the 2023 herbivory experiment. Dark solid interior lines represent mean growth of each genotype and the thin lines are the growth of individual caterpillars.

**Figure 2:**
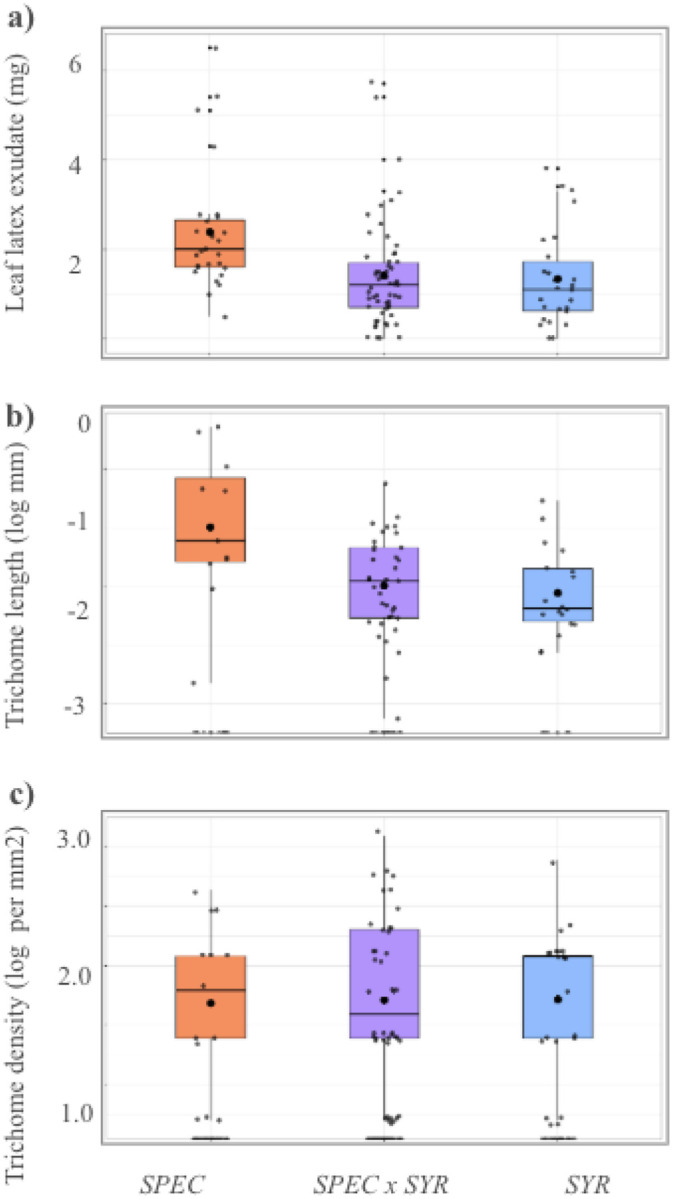
Monarch caterpillar growth measured over ten days in three separate experiments. The relative growth of monarch caterpillars reared on geographically wide-ranging parental species (orange; *A. speciosa*, blue; *A. syriaca*) is faster than growth of caterpillars on genetically diverse hybrids between the species (purple; *A. syriaca x A. speciosa)*. The three separate experiments conducted in **a)** 2021, **b)** 2023, and **c)** 2025 show the same pattern.

To determine if there were average growth differences among monarch caterpillars reared on *A. syriaca*, *A. speciosa*, and genetically diverse *A. syriaca x speciosa* hybrids, we measured caterpillar growth over a 10 day period. The growth of hybrid genotypes was slower, on average, than growth on the parental species (Fig. 1). Three independent experiments show the same qualitative pattern (Fig. 2), with hybrid-reared caterpillars growing more slowly than caterpillars reared on either parental species.

To compare monarch growth across host plant genotypes while accounting for variation among experiments, we fit a linear mixed-effects model with plant genotype group as a fixed effect and experiment as a random effect. Relative growth differed significantly among plant genotype groups when experiments were analyzed together, using growth values calculated within each experiment (F(2, 54.33) = 11.42, P = 0.000197). Model-estimated mean relative growth was lowest for caterpillars reared on hybrids (0.472), higher for those reared on *A. syriaca* (0.710), and highest for those reared on *A. speciosa* (0.745). Tukey-adjusted pairwise comparisons showed that caterpillars reared on hybrids grew significantly less than those reared on *A. speciosa* (β = 0.273 ± 0.068, *P* = 0.000179) or *A. syriaca* (β = 0.238 ± 0.063, *P* = 0.000391).

### Comparison of leaf cardenolide concentration in parental and genotypically diverse Asclepias syriaca x speciosa hybrids

**Figure 3:**
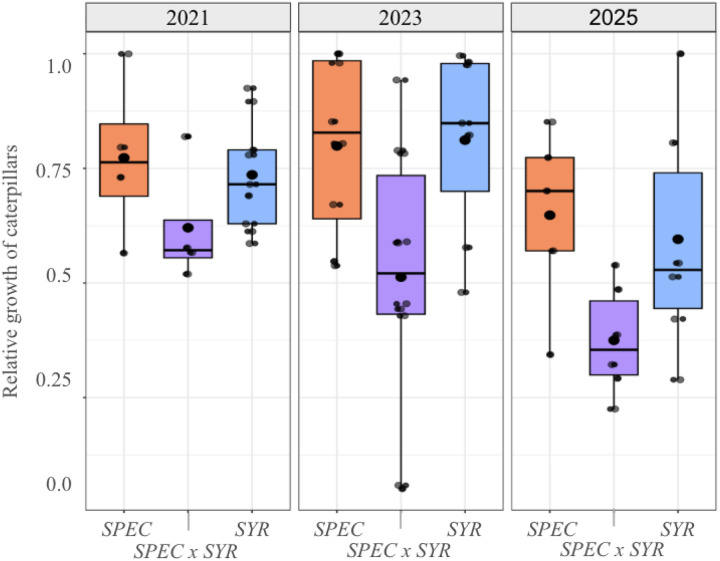
Leaf cardenolide expression measured in **a)** cardenolide richness, **b)** cardenolide concentration measured in milligram total cardenolides per gram of dry mass (DM), and **c)** polarity index. These metrics did not differ among genotype groups.

Despite significant differences in monarch caterpillar growth across plant genotype groups, we did not find that plant cardenolide chemistry differed significantly among genotype groups (*A. syriaca*, *A. speciosa*, and *Asclepias syriaca x speciosa)* in the summary metrics measured, including cardenolide richness (number of detected cardenolide peaks; Fig. 3a) total concentration in leaf tissue, total cardenolide concentration (Fig. 3b), or the total number of cardenolide peaks detected (Fig. 3c).

Plant cardenolide concentration between the genotype groups did not differ (linear mixed model, Type III ANOVA: F_2,23_ = 0.097, p = 0.908). Likewise, the plant polarity index did not differ among species (F_2,22.5_=1.36, p = 0.277, and plant peak richness did not differ (F_2,23_=0.056, p = 0.946). Tukey-adjusted pairwise comparisons were non-significant for all species contrasts in all three plant chemistry metrics (p > 0.306).

### Overall sequestration is lower in butterfly tissues reared on hybrid genotypes

To identify if the genotype group predicted the sequestration of cardenolides in the adult butterflies, we quantified the concentration in the forewings, thorax, and abdomens of monarchs reared on the three genotype groups. When summed across the wing, thorax, and abdomen tissues normalized cardenolide concentration differed significantly among host genotype groups (Type III ANOVA: F(2,24) = 3.63, p = 0.042). Estimated means were lowest for butterflies reared on hybrids and highest for butterflies reared on *A. speciosa*. Contrasts using hybrid as the reference indicated a suggestive difference between *A. speciosa*-hybrid after Dunnett correction (p = 0.052), whereas the *A. syriaca*-hybrid contrast was not significant (p = 0.182).

**Figure 4:**
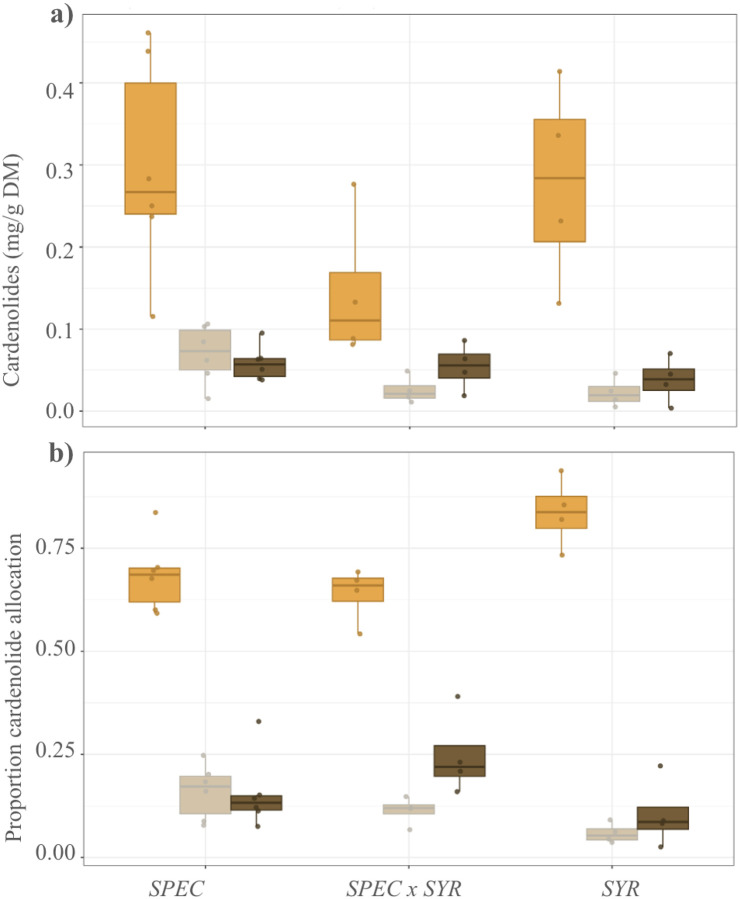
Total cardenolide concentration a) is significantly lower in monarchs reared on *A. syriaca x speciosa* hybrids (Type III ANOVA: F(2,24) = 3.63, p = 0.042), visualized here in wing (orange), thorax (tan), and abdomen (brown) tissues than on parental species. Allocation among tissues types b) is similar among genotype classes.

Although the total concentrations differed, the allocation of cardenolides among tissue forewing, abdomen, and thorax was similar among butterflies reared on the different genotype groups (Fig. 4a). Across all genotypes, wings contained significantly higher cardenolide concentrations than both thorax and abdomen (Tukey-adjusted p < 0.0001), while thorax and abdomen did not differ (p = 0.314) (Fig. 4b).

### Physical defense do not predict caterpillar growth

**Figure 5:**
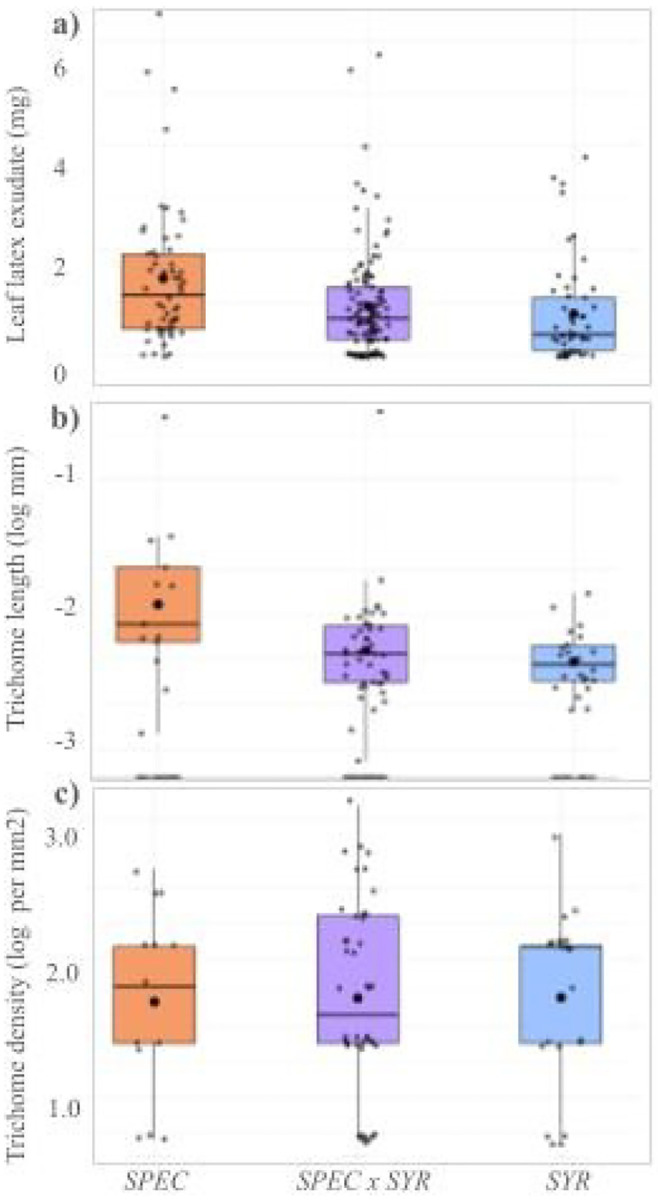
Plant physical defenses of a) leaf latex exudate, b) trichome length and c) density do not differ among genotype groups in 8–10 week old milkweed seedlings.

To determine whether leaf physical defenses (latex exudation and trichome length, and trichome density) different among genotype groups (Fig. 5), we compared genotype means with ANOVA. Latex exudation differed among genotypes in the full model (F(2,214) = 5.81, p = 0.0035) and post hoc comparisons showed that *A. speciosa* exuded significantly more latex, on average, than *A. syriaca* (t = 3.10, p = 0.006) and hybrid genotypes (t = 2.911, p = 0.011). There was no significant difference in a comparison of hybrid genotypes and *A. syriaca* (t = 0.746, p = 0.736). In contrast trichome length (F(2,213) = 0.21, p = 0.810) and trichome density did not differ significantly among genotype groups (F(2,213) = 1.80, p = 0.169).

### Manganese (Mn) concentration is negatively related to caterpillar growth and sequestration

In a separate cohort of plants (not included in any herbivore growth experiment) we measured plant nutrient content of the same plant families included in the herbivory experiments. Across 14 nutrient traits, sulfur, iron, and manganese showed the clearest evidence of genotype-associated differences. Sulfur differed most strongly among genotype classes and remained significant after Benjamini-Hochberg correction. However, its concentration is unrelated to caterpillar growth.

Iron and manganese also remained significant after correction. Although several other nutrients showed raw p-values below 0.05, including nitrogen, phosphorus, potassium, magnesium, calcium, and C:N ratio, these did not remain significant after multiple-testing correction. Thus, the strongest evidence for nutrient differences among genotype classes was concentrated in sulfur, iron, and manganese, rather than reflecting a broad shift across all measured nutrients.

We observed a negative relationship between plant manganese concentration and caterpillar relative growth. Caterpillars reared on plants with higher manganese concentrations tended to have lower relative growth rates, though this correlation was marginally non-significant (Pearson’s r = −0.50, df = 14, p = 0.051).

## DISCUSSION

Monarchs reared on hybrid milkweeds (*A. syriaca x speciosa*) grew more slowly than monarchs reared on either parental species, and this reduction in growth was qualitatively consistent across three independent experiments. In addition to reduced larval performance, adults that developed on hybrid plants had lower total cardenolide concentrations across adult tissues, indicating reduced sequestration of host-derived chemical defenses. Neither plant cardenolide metrics nor measured physical defense traits clearly mirror expected patterns of monarch growth and adult sequestration. However, we find that hybrid milkweeds produce higher manganese concentrations than the parental species and plant families with higher manganese tended to support slower caterpillar growth, consistent with the possibility that altered micronutrient accumulation in hybrid plants affects host quality. These results suggest that hybridization influenced not only monarch performance on host plants, but also the extent to which monarchs acquired plant-derived chemical defense

The lower adult cardenolide concentration we observe in monarchs reared on *A. syraica x speciosa* hybrids is potentially biologically important as monarchs use cardenolides in their own defense (Brower and Glazier 1975; Malcolm and Brower 1989). To our knowledge, hybridization has often been studied for its effects on plant chemistry and herbivore performance (Cheng *et al*. 2011; Kirk *et al*. 2012; Caseys *et al*. 2012; Renoult *et al*. 2025), but its consequences for host-derived acquired defense in herbivores remain largely unresolved.. In monarchs, sequestered cardenolides can influence interactions with multiple classes of natural enemies, including avian predators (Malcolm and Brower 1989), *Pteromalus* parasitoid wasp species (Stenoien *et al*. 2019), and the protozoan parasite *O. elektroscirrha* (Gowler *et al*. 2015; Hoang *et al*. 2017; de Roode and Hunter 2019; Hoogshagen *et al*. 2023), with parasitoid wasps and protozoan parasites being particularly sensitive to concentration of cardenolides. Lower adult cardenolide burdens in monarchs reared on hybrids could therefore reduce protection against enemies and could be maladaptive. Although we did not measure predation, parasitism, or parasite resistance, the observed reduction in adult cardenolide content may have downstream ecological consequences.

The absence of clear differences among host genotype groups in plant summary cardenolide chemistry highlight that the metrics measured here are either too coarse or cardenolides are not the driving factor in reduced caterpillar performance. Particular cardolide mixtures found in plants are sequestered by herbivores to balanced physiological cost of sequestration and toxicity to predators (Agrawal *et al*. 2021, 2024; Hoogshagen *et al*. 2023), potentially amplifying the effect of altered sequestration in monarchs. Monarchs use many species with diverse cardenolide mixtures and toxicity as host plants (Züst *et al*. 2019), yet hybrid plants could produce untested individual cardenolides or mixtures.

Plant hybridization often alters herbivore resistance, susceptibility, and performance in variable ways. Studies across several systems have shown that hybrids can express intermediate, transgressive, or novel chemical phenotypes and that herbivore outcomes may differ accordingly (Cheng *et al*. 2011; Kirk *et al*. 2012; Caseys *et al*. 2012; Renoult *et al*. 2025). Other studies have shown that herbivore performance on hybrids can differ across life-history stages, such that oviposition, larval growth, and survival are not necessarily controlled by the same plant traits (Torp *et al*. 2013). Our results are consistent with these findings and add that hybrid host-plants may also modify herbivore-acquired defense. In naturally occurring hybrid zones, this may be especially relevant as herbivores encounter hybrid swarms rather than the host species to which they are adapted.

The lack of clear differences in plant summary chemistry may indicate the need for further analysis into interactions among defenses in *A. syriaca x speciosa* hybrids. This is consistent with growing evidence that milkweed defenses operate as integrated combinations rather than as independent axes. Latex, trichomes, cardenolides, leaf structural traits, can act synergistically, antagonistically, or in context-dependent ways, and these interactions may be more important to herbivores than any single trait measured in isolation (Rasmann *et al*. 2009; Jones and Agrawal 2019; Edwards *et al*. 2023). In milkweed specifically, defense traits do not always co-vary in simple ways, and combinations of traits can shape herbivore behavior and performance more strongly than single traits alone (Agrawal *et al*. 2014; Craig and Agrawal 2025). Thus, the differences in monarch growth and sequestration could be due to specific constellations of defensive traits found in the highly variable *A. syriaca x speciosa* hybrids. These shuffled defensive traits, rather than simply shifting trait means, may produce herbivore responses that are difficult to predict from parental averages or from one trait at a time.

Beyond plant defenses, altered hybrid nutritional quality impacts herbivore performance. Altered trace metal uptake in plants has important effects on herbivores (Ben-Shahar 2018) and the negative relationship between plant manganese concentration and monarch growth and sequestration could suggest that reduced monarch performance on hybrids may not be driven by plant defensive traits. Manganese is an essential co-factor required for cellular function (Iqbal *et al*. 2025). Chronic dietary exposure, however, has been shown to have many negative effects on other insects (Ternes *et al*. 2014; Martinek *et al*. 2018), including other *Lepidoptera* species (Kula *et al*. 2014). In *Drosophila melanogaster*, exposure to excessive manganese leads to increased mortality and impaired locomotor performance (Oboh *et al*. 2018). Although the two- to three-fold increase in manganese in hybrid plants is consistent with this possibility, additional work is needed to determine whether manganese directly contributes to reduced monarch performance or reduced cardenolide sequestration.

Although the cause of reduced monarch performance is unknown, our work holds implications of the impacts of plant hybridization on specialist insect herbivores, especially those that sequester plant toxins. Identifying the cause of reduced monarch performance on hybrids will require pairing plant and butterfly chemistry at the individual metabolite level. Our work here sampled a highly variable set of milkweed genotypes, even within each of the parental species, hindering our ability to link individual metabolic or physical traits to the effect in monarchs. We also did not identify individual cardenolides with mass-spectrometry and thus there is a possibility that hybrid milkweeds produce cardenolides that subvert typical detoxification. Further studies will interrogate the specific identity of cardenolide toxin in the plants and monarchs, identifying whether there are unique combinations of cardenolides, where sequestration breaks down, how plant nutrition affects growth, or whether specific combinations of hybrid plant traits impact performance.

## Supplementary Material

Supplementary Files

This is a list of supplementary files associated with this preprint. Click to download.

- SupplementaryTablesandFigures1.pdf
- SupplementalDataTable1.xlsx
- SupplementalDataTable2.xlsx
- SupplementalTable3.xlsx

## Figures and Tables

**Table 1:** Leaf tissue elemental composition of sulfur, iron, and manganese differ among milkweed genotypes. Values are genotype means with standard deviations for *A. speciosa*, hybrids, and *A. syriaca*. For each element, genotype differences were tested using linear mixed models (LMM) with genotype as a fixed effect and family as a random effect. P-values for the overall genotype effect were adjusted across elements using the Benjamini-Hochberg false discovery rate correction. Significant Tukey-adjusted post hoc pairwise comparisons are reported when the overall genotype effect was significant.

Element		*A. speciosa (SPE)*	*A. syriaca x speciosa (HYB)*	*A. syriaca (SYR)*		
	Unit	mean ± sd	mean ± sd	mean ± sd	LMM p-value	Significant pairwise comparison(s)
Calcium	% rate	1.86 ± 0.29	1.80 ± 0.40	1.41 ± 0.40	0.25	
Carbon	% rate	42.19 ± 0.16	43.37 ± 1.21	44.11 ± 0.89	0.06	
Magnesium	% rate	0.47 ± 0.08	0.39 ± 0.05	0.36 ± 0.05	0.20	
Nitrogen	% rate	3.38 ± 0.48	3.86 ± 0.70	3.20 ± 0.47	0.57	
Phosphorus	% rate	0.41 ± 0.07	0.60 ± 0.26	0.40 ± 0.11	0.67	
Potassium	% rate	4.33 ± 0.63	3.77 ± 0.96	3.28 ± 0.54	0.48	
Sodium	% rate	0.006 ± 0.003	0.005 ± 0.005	0.005 ± 0.005	1	
Sulfur	% rate	1.37 ± 0.12	0.67 ± 0.18	0.56 ± 0.15	**0.001**	SPE-SYR
Boron	ppm	42.15 ± 13.77	56.11 ± 26.59	33.33 ± 11.53	0.96	
Copper	ppm	2.00 ± 0.00	2.57 ± 1.04	2.77 ± 1.63	1	
Iron	ppm	73.25 ± 11.12	91.44 ± 13.88	72.95 ± 7.99	**0.04**	SPE-HYB; HYB-SYR
Manganese	ppm	154.42 ± 61.82	291.14 ± 119.08	170.51 ± 45.61	**0.02**	SPE-HYB; HYB-SYR
Zinc	ppm	67.40 ± 21.60	87.67 ± 34.46	83.62 ± 51.29	1	
C/N Ratio		12.76 ± 1.80	11.94 ± 2.96	14.57 ± 2.26	0.65	

## Data Availability

All data supporting the findings of this study are available within the paper and its Supplementary Information
